# Radicals and Ions Formed in Plasma-Treated Organic Solvents: A Mechanistic Investigation to Rationalize the Enhancement of Electrospinnability of Polycaprolactone

**DOI:** 10.3389/fchem.2019.00344

**Published:** 2019-05-16

**Authors:** Silvia Grande, Francesco Tampieri, Anton Nikiforov, Agata Giardina, Antonio Barbon, Pieter Cools, Rino Morent, Cristina Paradisi, Ester Marotta, Nathalie De Geyter

**Affiliations:** ^1^Research Unit Plasma Technology, Department of Applied Physics, Faculty of Engineering and Architecture, Ghent University, Ghent, Belgium; ^2^Department of Chemical Sciences, Università degli Studi di Padova, Padua, Italy

**Keywords:** non-thermal plasma (NTP), plasma jet in liquid, chloroform, dimethylformamide (DMF), spin-trapping

## Abstract

This paper reports and discusses the beneficial effects on the quality of electrospun polycaprolactone nanofibers brought about by pretreatment of the solvent with non-thermal plasma. Chloroform/dimethylformamide 9:1 (CHCl_3_:DMF 9:1) and pure chloroform were pretreated by a few minute exposure to the plasma generated by an atmospheric pressure plasma jet (APPJ). Interestingly, when pure chloroform was used, the advantages of plasma pretreatment of the solvent were way less pronounced than found with the CHCl_3_:DMF 9:1 mixture. The chemical modifications induced by the plasma in the solvents were investigated by means of complementary analytical techniques. GC-MS revealed the formation of solvent-derived volatile products, notably tetrachloroethylene (C_2_Cl_4_), 1,1,2,2-tetrachloroethane (C_2_H_2_Cl_4_), pentachloroethane (C_2_HCl_5_), hexachloroethane (C_2_Cl_6_) and, in the case of the mixed solvent, also N-methylformamide (C_2_H_5_NO). The chlorinated volatile products are attributed to reactions of ·Cl and Cl-containing methyl radicals and carbenes formed in the plasma-treated solvents. ·Cl and ·CCl_3_ radicals were detected and identified by EPR spectroscopy analyses. Ion chromatography revealed the presence of Cl^−^, ${\text{NO}}_{3}^{-}$, and HCOO^−^ (the latter only in the presence of DMF) in the plasma-treated solvents, thus accounting for the observed increased conductivity and acidification of the solvent after plasma treatment. Mechanisms for the formation of these solvent derived products induced by plasma are proposed and discussed. The major role of radicals and ions in the plasma chemistry of chloroform and of the chloroform/dimethylformamide mixture is highlighted. The results provide insight into the interaction of plasma with organic solvents, a field so far little explored but holding promise for interesting applications.

## Introduction

It was recently reported that the application of non-thermal plasma leads to remarkable improvements in the electrospinnability of polymer solutions to form nanofibers (Shi et al., 2011; Colombo et al., 2014; Grande et al., 2017; Rezaei et al., 2018b) but no explanation was given for the origin of these effects.

Electrospinning is an efficient and powerful fabrication process to obtain high quality nanofibers with a wide range of diameters, from several micrometers down to a few tens of nanometers (Frenot and Chronakis, 2003; Reneker and Yarin, 2008). Because of its ease of use and versatility, this technique has led to an exponential increase in the production of nanofibers and their application in many different fields such as filtration (Shabafrooz et al., 2014), sensors (Huang et al., 2003), electronics (Long et al., 2012), and biomedicine (Venugopal and Ramakrishna, 2005). A typical electrospinning process involves the application of a high voltage (HV) between a tip, from which the polymer solution is extruded, and a collector (Bhardwaj and Kundu, 2010). The HV acting on free charges present in the polymer solution attracts the liquid toward the collector generating a jet which upon evaporation of the solvent leads to the formation of fibers on the collector itself (Teo and Ramakrishna, 2006). Various parameters influence the properties and quality of electrospun nanofibers, a major role being played by the characteristics of the polymer solution, notably its electrical conductivity, viscosity and surface tension. Indeed, a commonly used strategy to enhance the electrospinnability of polymer solutions consists in increasing the solution conductivity with additives such as salts or polar organic solvents (Hsu and Shivkumar, 2004; Qin et al., 2007; Ryu and Kwak, 2013). However, the use of additives can affect the chemical composition and properties of electrospun nanofibers and pose safety and environmental issues (Zong et al., 2002; Hsu and Shivkumar, 2004).

Non-thermal plasma is a partially ionized gas in non-equilibrium thermal state in which the electrons temperature is much higher than that of ions and neutrals. Such plasmas are conveniently generated by electrical discharges in a gas at room temperature and atmospheric pressure. When applied in contact with liquids, the discharges generate intense UV radiation, shock waves and active radicals, which can induce variations of the chemical composition of the liquid itself as well as directly affect any organic or biological material present in the system (Bruggeman and Leys, 2009; Bruggeman et al., 2016). Many electrode configurations and experimental set-ups have already been employed to work with liquids (Bruggeman et al., 2016), but only a few studies involve organic solvents for polymer solution modification (Shi et al., 2011; Colombo et al., 2014; Grande et al., 2017; Rezaei et al., 2018b).

In previous work by some of the authors of this paper, an atmospheric pressure plasma jet (APPJ), explicitly designed to ensure a close and intense contact between the plasma plume and the liquid (Grande et al., 2017; Rezaei et al., 2018a,b), was used to treat solutions of polycaprolactone (PCL) or polylactic acid (PLA) in solvent mixtures of chloroform (CHCl_3_) and N,N-dimethylformamide (DMF) (Grande et al., 2017; Rezaei et al., 2018b). It was found that plasma treatment of these solutions before electrospinning leads to nanofibers of better quality, i.e., with a bead-free morphology and uniform diameter, than obtained in control experiments without plasma. The analysis of the polymers by size exclusion chromatography (SEC) and X-ray photoelectron spectroscopy (XPS) showed that the molecular weight and the surface chemical composition of electrospun PCL nanofibers were not significantly affected by the APPJ treatment. Significant changes were instead observed in some important solution properties, notably conductivity and viscosity, both of which were found to increase after plasma treatment, and pH, which instead decreased. Thus, the enhanced electrospinnability was mainly attributed to these modifications.

Analogous improvements in the quality of electrospun fibers were obtained by plasma pretreatment of PLA solutions (Rezaei et al., 2018a,b). Interestingly, some improvement was also observed when the pure solvent, or solvent mixture, was treated with plasma prior to the addition of PLA (Rezaei et al., 2018a). Building on these promising results we studied the behavior of PCL in two organic solvents, pure chloroform and CHCl_3_:DMF 9:1 mixture, with the dual objective of verifying the scope and generality of the phenomenon observed for PLA and, more importantly, of studying in detail what happens to the organic solvent when it is treated with plasma. The latter subject is of great interest *per se*, as our present knowledge of the interaction between plasma and organic solvents and of its outcomes is very limited. Analyses were thus performed both on the nanomaterials produced and on the solvents. The quality and morphology of electrospun PCL nanofibers obtained according to various experimental protocols were investigated by means of SEM analysis, while plasma treated solvents were analyzed by gas chromatography coupled with mass spectrometry (GC-MS), by EPR spectroscopy, with the use of spin traps, and by ion chromatography, to gather information on the ions formed in the plasma-treated solvents. The combination of these techniques provided a powerful diagnostic array to gain insight into the complex mechanisms induced by plasma treatment. Comparison of the results obtained with pure CHCl_3_ and with a CHCl_3_:DMF 9:1 mixture turned out to be particularly informative.

## Materials and Methods

### Materials

PCL pellets (M_n_ = 80,000 g/mol), chloroform (CHCl_3_, > 99%), N,N-dimethylformamide (DMF, > 99%) N-tert-butyl-α-phenylnitrone (PBN, 98%), sodium carbonate (Na_2_CO_3_), sodium bicarbonate (NaHCO_3_), sodium nitrate (NaNO_3_), potassium chloride (KCl), and formic acid (HCOOH) were purchased from Sigma-Aldrich and used without further purification. Argon gas (Alphagaz 1) was purchased from Air Liquide. Ultrapure grade water (milliQ water) was obtained by filtration of deionized water with a Millipore system.

### APPJ Treatment of PCL Solvents

The plasma source used in this work to treat PCL solvents is an APPJ specifically designed for liquid treatment. The set-up was already described in detail in a previous work (Grande et al., 2017). In short, the plasma was generated inside a thin quartz capillary fed by an argon flow. A tungsten needle was placed within the capillary and acted as high-voltage electrode, while a ring-shaped copper grounded electrode was placed around the quartz capillary at 4.5 cm from the tip of the tungsten needle. A constant argon flow of 1 standard liter per minute (slm) was sent through the capillary. Successively, the discharge was ignited by applying an AC high voltage (fixed frequency of 50 kHz) to the high-voltage electrode with a peak-to-peak value of 7.6 kV. A small reactor chamber, which can contain the solvents, was placed on top of the capillary exit by fixing a quartz tube with an inside and outside diameter of 13 mm and 20 mm respectively to a stainless-steel flange possessing a small opening where the APPJ quartz capillary can be inserted. The distance between the top of the grounded electrode and the bottom of the stainless-steel flange was maintained at 0.5 cm to ensure electrical isolation. For all experiments, a fixed liquid sample volume of 10 mL was introduced into the reactor chamber using a glass syringe. Afterwards, the top of the reactor chamber was covered with a stainless-steel flange containing a small opening of 2 mm acting as gas outlet, thereby limiting solvent evaporation during plasma treatment. In this work, pure CHCl_3_ and a mixture of CHCl_3_:DMF (9:1 v/v) were exposed to the APPJ for a fixed plasma exposure time of 3 min. This treatment time was chosen based on the results obtained in plasma treatment of the polymer solutions (Grande et al., 2017). Under those conditions, it was observed that extending the treatment time beyond 3 min did not bring any further improvement on the morphology of electrospun nanofibers. Thus, the same treatment time was applied in this study to allow for a direct comparison of the results obtained with the two plasma activation protocols.

To electrically characterize the plasma, the voltage applied to the needle electrode was measured using a high voltage probe (Tektronix P6015A) while the charge on the electrodes was obtained by measuring the voltage over a capacitor of 10 nF placed in series with the grounded electrode. The obtained voltage-vs.-charge plot was visualized using a PC oscilloscope (Picoscope 3204A) enabling the construction of a Lissajous figure. From the area enclosed by this figure, the electrical energy consumed per voltage cycle E_el_ could be estimated. The electrical power P_el_ was then obtained by multiplying the electrical energy with the frequency of the feeding voltage, which is equal to 50 kHz in this work, and was found to be 4.8 W.

The Ar streamed samples, used as control, were prepared under the same conditions as the plasma treated samples except for the fact that plasma was turned off. After 3 min of Ar streaming of the mixture CHCl_3_:DMF 9:1 the remaining liquid volume was 8 mL.

### Preparation and Electrospinning of PCL Polymer Solutions

Five percent w/v PCL polymer solutions were prepared by dissolving PCL pellets in pristine and plasma-treated CHCl_3_ as well as the pristine and plasma-treated CHCl_3_:DMF mixture. Subsequently, the differently prepared PCL solutions were stirred at room temperature for 3 h and electrospun. In this study, a bottom-up electrospinning process was performed using a customized Nanospinner 24 electrospinning machine (Inovenso, Turkey). In a first step, the PCL polymer solution under study was loaded into a 5 mL standard syringe connected to a blunt-ended copper needle and placed into a syringe pump (NE-300 Just Infusion™ syringe pump). This syringe pump controlled the flow rate of the polymer solution through a polyethylene tube (inner diameter: 2 mm) ending in an aluminum pipe containing a single brass nozzle with an inner diameter of 0.8 mm. During the electrospinning process, the flow rate of the polymer solution was maintained at 0.1 mL/min. The metallic nozzle was placed vertically below a rotating stainless-steel collector (100 rpm) at a distance of 20 cm. During electrospinning, a DC high voltage of 30 kV was supplied to the nozzle, while the rotating cylinder was grounded. PCL nanofibers were subsequently collected on an aluminum sheet placed on top of the collecting cylinder.

### Characterization of the Electrospun PCL Nanofibers by SEM

The surface morphology of the PCL nanofibers was imaged using a JEOL JSM-6010 PLUS/LV scanning electron microscope (SEM). SEM images were acquired with an accelerating voltage of 5 or 7 kV, after coating the samples with a thin layer of gold making use of a sputter coater (JFC-1300 autofine coater, JEOL).

### Chemical Characterization of Pristine and Plasma-Treated Solvents

#### GC-MS

GC-MS analyses of the PCL liquid solvents under study (CHCl_3_ and a 9:1 mixture of CHCl_3_:DMF) before and after plasma treatment were carried out with an Agilent Technologies instrument (GC System 6850 Series, Mass Selective Detector 5973) using an HP-5ms column (30 m × 0.25 mm internal diameter). One microliter samples were injected and analyzed with the following temperature program: 50°C for 5 min, 50-200°C at 15°C/min and 200°C for 5 min.

#### Ion Chromatography, Conductivity and pH

To quantify the ionic species in the liquid solvents, 5 mL of MilliQ water was added to 5 mL solvent into a separating funnel. Afterwards, the aqueous phase was analyzed by ion chromatography using a Dionex-ICS-900 instrument equipped with a Dionex IonPac AS22 column. A mixture of 4.5 mM Na_2_CO_3_ and 1.4 mM NaHCO_3_ was used as eluent at a flow rate of 1.2 mL/min. Standard solutions of KCl and NaNO_3_ were used to obtain calibration lines for chloride and nitrate ions, respectively.

The conductivity of the aqueous phase was also determined using a FiveEasy™ conductivity meter (Mettler Toledo) equipped with an InLab720 conductivity probe operating in a conductivity range of 0.1 to 500 μS/cm, while the pH of the aqueous phase was obtained making use of a FiveEasy™ pH meter equipped with an InLab Science Pro-ISM pH probe.

#### Spin-Trapping Experiments

The spin-trapping measurements have been performed at room temperature using an X-band Bruker ELEXSYS spectrometer equipped with an ER 4103TM cylindrical mode resonator for aqueous and high-dielectric samples. In a first step, a solution of PBN 1.0·10^−2^ M was prepared in chloroform and subsequently treated in the plasma reactor for 3 min. Immediately after plasma treatment, the solution was transferred to an EPR flat cell (500 μL capacity) and rapidly introduced in the EPR spectrometer. EPR spectra were collected at room temperature, at different delays after the introduction of the sample in the spectrometer, in order to follow the time evolution of the EPR signals; each spectrum was the average of 10 scans. The acquisition parameters were: modulation frequency 100 kHz, scan range 100 G, modulation amplitude 1.5 G, receiver gain 60 dB, microwave frequency 9.77 GHz (scaling of the field has been used), power attenuation 18 dB, time constant, 5.12 ms, scan time 41.94 s, conversion time 40.96 ms. All EPR spectra have been reproduced using EPR WinSim software in order to isolate and identify all the radical species (Duling, 1994).

## Results

### SEM Analysis of Electrospun PCL Nanofibers

**Figure 1:**
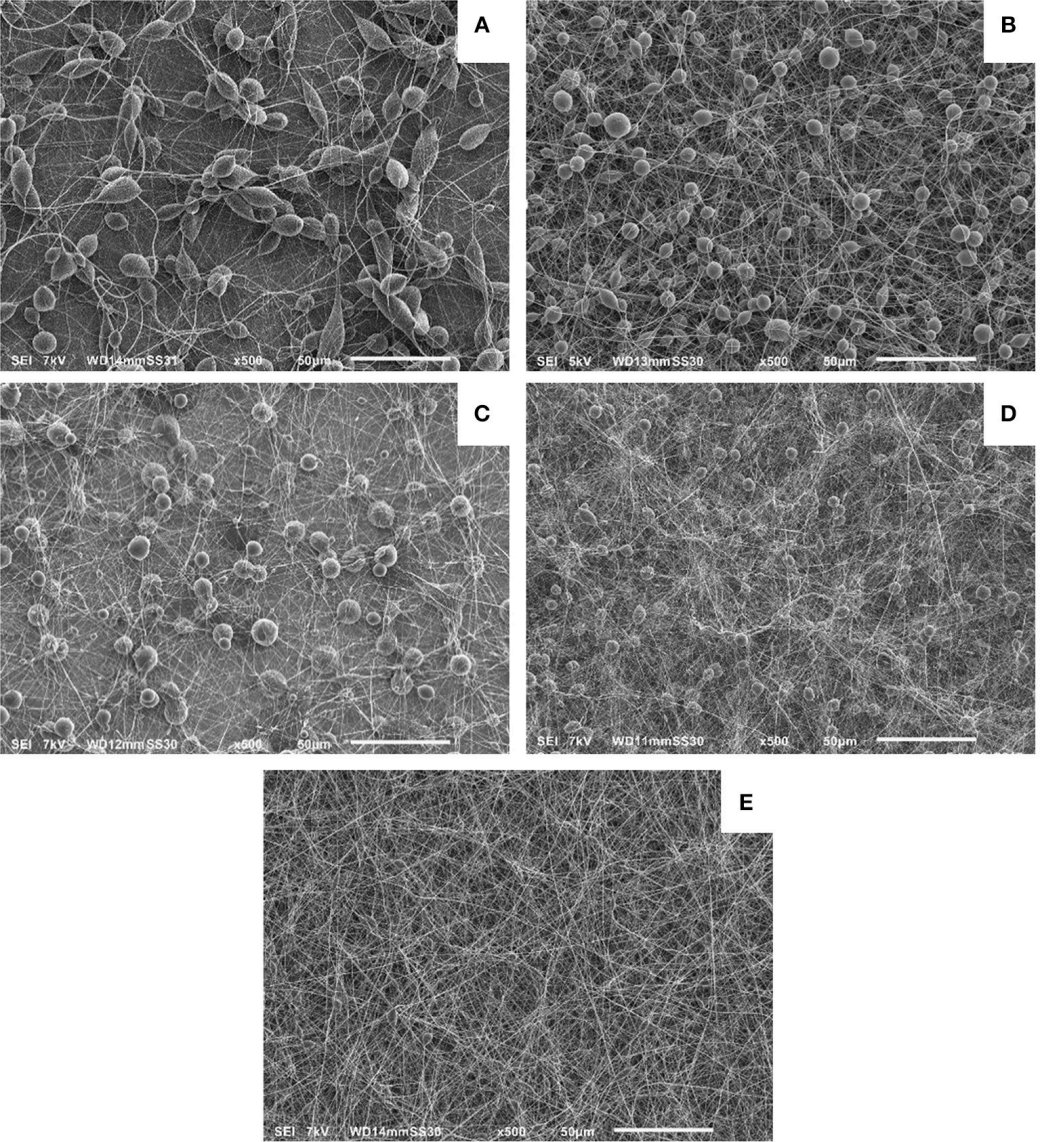
SEM images of different electrospun PCL polymer solutions: PCL dissolved in untreated CHCl_3_ **(A)**; PCL dissolved in plasma-treated CHCl_3_ **(B)**; PCL dissolved in untreated CHCl_3_:DMF 9:1 **(C)**; PCL dissolved in argon-streamed CHCl_3_:DMF 9:1 **(D)**; PCL dissolved in plasma-treated CHCl_3_:DMF 9:1 **(E)**.

The APPJ (Grande et al., 2017), briefly described in the Experimental Section, was used to treat the pure solvents (CHCl_3_ and the CHCl_3_:DMF 9:1 mixture), after which PCL was dissolved in the plasma-treated solvents. The obtained PCL solutions were subsequently electrospun and SEM analyses were carried out of electrospun PCL nanofibers obtained under different conditions. Figure 1 reports SEM images obtained for these experiments and for controls run without plasma pretreatment of the solvents. Specifically Figures 1A,B show the SEM images of electrospun fibers obtained when PCL was dissolved in pristine and in plasma-treated CHCl_3_, respectively. In both cases, non-uniform PCL fibers with a large amount of beads can be observed, but the average size of the beads appears to be smaller in the plasma treated samples. The SEM images relative to the electrospun PCL fibers obtained when PCL was dissolved in untreated and plasma-treated CHCl_3_:DMF 9:1 are reported in Figures 1C,E, respectively.

These images clearly reveal the dramatic improvement in the nanofibers quality achieved by plasma treatment of the solvent preliminary to addition of PCL and electrospinning of the solution. The obtained sample consisted of a uniform and almost bead-free mesh. A control experiment was carried out to determine the possible contribution to the effects observed in Figure 1E by modifications of the solvent composition due to vapor stripping by the argon flow used to sustain the discharge during plasma treatment. Figure 1D shows a SEM image of the sample obtained in this experiment, which was carried out with the plasma switched off and flowing argon for a time long enough to achieve the same volume reduction as obtained in experiments with “plasma on.” It is seen that without plasma, non-uniform PCL fibers containing a large number of beads were obtained. However, the beads are definitely smaller compared to those in the untreated control sample (Figure 1C). This improvement can be ascribed to the different ratio of the two solvents in the final solution which was used to electrospin the fibers shown in Figure 1D. This effect is the result of the two solvents different evaporation rates and the consequent increase in the relative amount of DMF with respect to the 9:1 ratio in the original mixture.

### Chemical Analysis of Plasma-Treated Solvents

#### GC-MS Analyses

**Figure 2:**
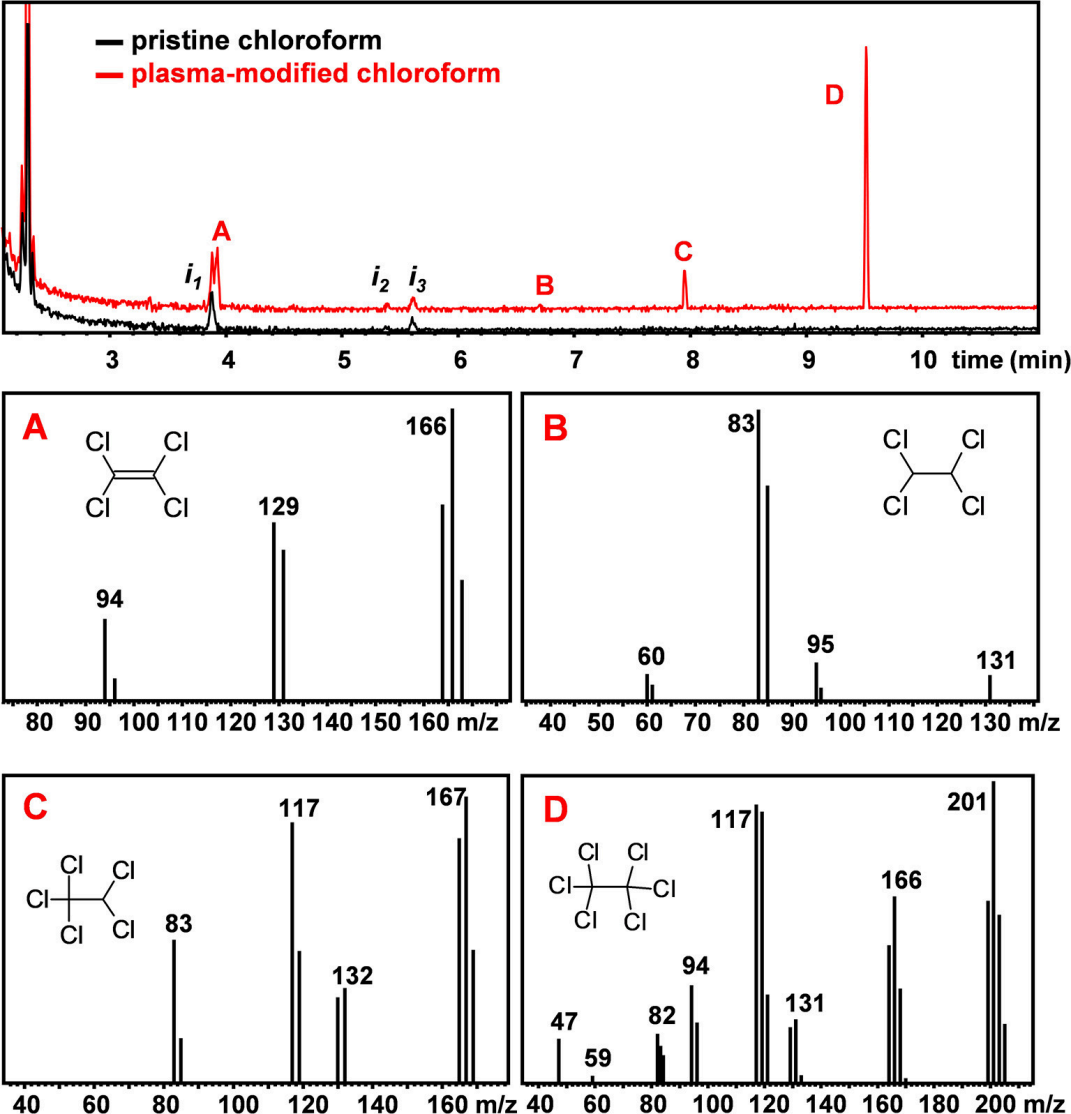
GC-MS chromatogram of pristine (black) and plasma-treated (red) CHCl_3_. MS spectra of peaks A to D. The labeled peaks were identified to be **(A)**: tetrachloroethylene (C_2_Cl_4_), **(B)** 1,1,2,2-tetrachloroethane (C_2_H_2_Cl_4_), **(C)** pentachloroethane (C_2_HCl_5_) and **(D)** hexachloroethane (C_2_Cl_6_).

To investigate possible modifications of the solvent composition and the formation of new volatile organic compounds due to the plasma treatment, GC-MS analyses of untreated and plasma-treated CHCl_3_ and CHCl_3_:DMF (9:1) were performed. Figure 2 reports the chromatograms of CHCl_3_ before and after 3 min plasma treatment, respectively. In the chromatogram of untreated CHCl_3_ some impurities were detected and are labeled as i_n_. After CHCl_3_ was treated with plasma for 3 min, four additional peaks (A to D) were detected in the chromatogram. Based on the analysis of their mass spectra, reported in Figure 2, and comparison with reference spectra (Linstrom and Mallard, 2019), these additional peaks could be ascribed to chlorinated ethanes (1,1,2,2-tetrachloroethane, pentachloroethane, and hexachloroethane) and tetrachloroethylene. The anomalous isotopic distribution observed in some of our spectra are due to low signal intensities.

**Figure 3:**
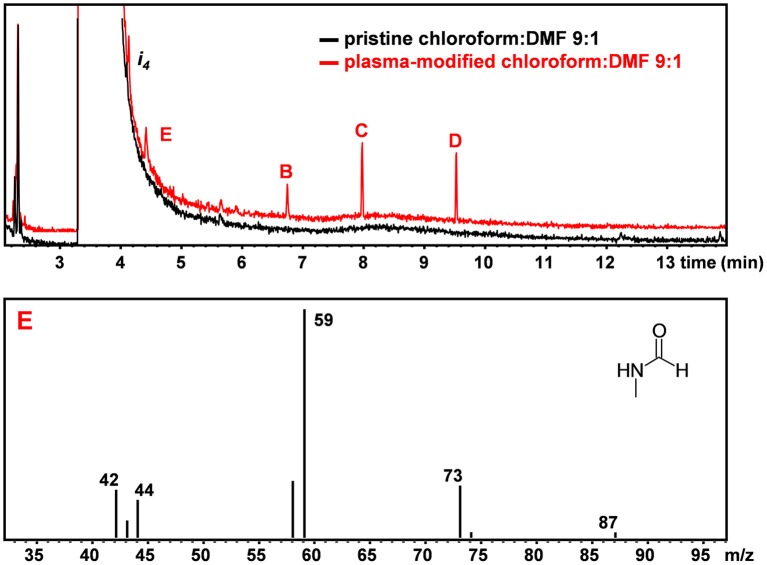
GC-MS chromatogram of pristine (black) and plasma-treated (red) CHCl_3_:DMF. MS spectrum of peak E leading to the identification of N-methyl-formamide (C_2_H_5_NO).

Figure 3 shows the chromatograms of the pristine and plasma-treated CHCl_3_:DMF 9:1 mixture, respectively. Figure 3 shows an additional impurity found in DMF, labeled as i_4_, which could be identified as formamide from its mass spectrum. In the chromatogram of plasma-treated CHCl_3_:DMF 9:1, the same peaks as detected in the chromatogram of plasma-treated CHCl_3_ can be observed, except for the peak attributed to tetrachloroethylene, which was hidden by the broad peak due to DMF. Moreover, the relative ratio between the peaks of the three chloroethanes (B, C, and D) was different with respect to the case in which these were formed by plasma treatment of pure CHCl_3_. In particular, hexachloroethane was no longer the major chloroethane formed and the relative amount of 1,1,2,2-tetrachloroethane was significantly higher. An additional peak was also detected in the chromatogram of plasma-treated CHCl_3_:DMF (peak E in Figure 3), which could be attributed, on the basis of its MS spectrum, to N-methylformamide.

#### Ion Chromatography, Conductivity and pH

As ascertained in previous publications (Šunka et al., 1999; Rezaei et al., 2018a,b), the plasma treatment of polymer solutions induces an increase in electrical conductivity of the liquids. In this work, we investigated whether this increase also occurs when the solvents are treated by plasma in the absence of polymers. Table 1 shows the conductivity of the aqueous phase used for the extraction of water soluble species from the treated solvents (as described in the experimental part). For pure CHCl_3_ a large increase in conductivity was indeed observed after plasma treatment. In the case of the CHCl_3_:DMF 9:1 solvent mixture, a tremendous increase in conductivity was induced by plasma treatment of the pristine mixture. In contrast, only a slight increase was observed after argon streaming of CHCl_3_:DMF (Table 1), confirming that the increase in conductivity was really due to plasma treatment. To identify and quantify the ions responsible for the solution conductivity, ion chromatography was applied.

**Table 1:** Extracted water: conductivity, pH and concentration of chloride and nitrate from different solvents.

**Sample**	**Conductivity (μS/cm)**	**pH**	**[Cl^**−**^] (mM)**	**[${\text{NO}}_{3}^{-}$] (mM)**
CHCl_3_ untreated	2.3 ± 0.2	4.3 ± 0.2	0.13 ± 0.04	n./a.
CHCl_3_ plasma-treated	52.0 ± 1.0	3.4 ± 0.2	0.5 ± 0.2	0.020 ± 0.014
CHCl_3_ + DMF untreated	5.7 ± 0.5	4.0 ± 0.2	0.20 ± 0.04	n./a.
CHCl_3_ + DMF Ar-streamed	7.3 ± 0.5	4.8 ± 0.2	0.20 ± 0.04	n./a.
CHCl_3_ + DMF plasma-treated	328 ± 5	2.5 ± 0.2	4.9 ± 0.7	0.019 ± 0.009

**Figure 4:**
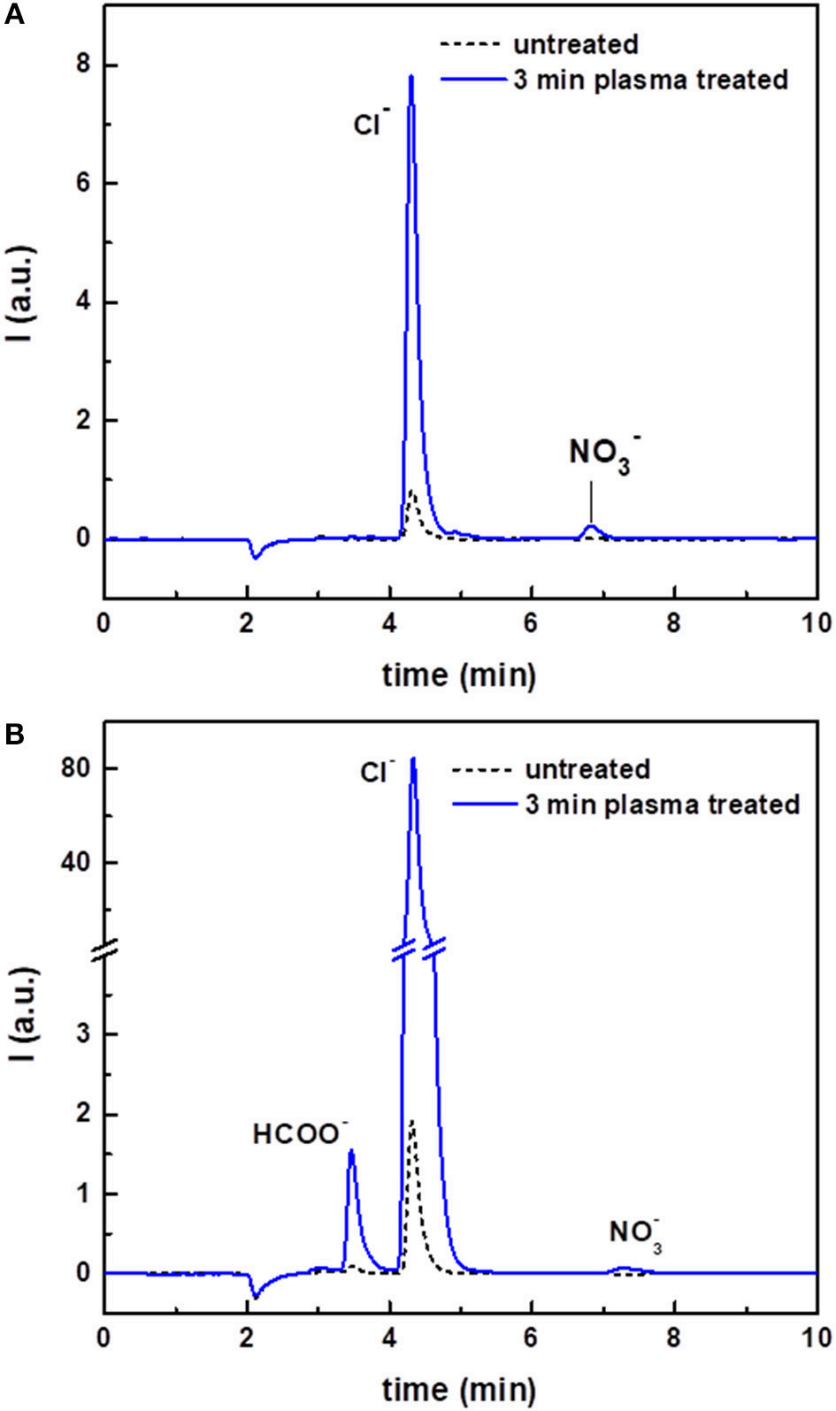
Ion chromatograms of untreated and plasma-treated CHCl_3_ **(A)** and of untreated and plasma-treated CHCl_3_:DMF 9:1 **(B)**.

The ion chromatograms of untreated and plasma-treated chloroform are shown in Figure 4A. The untreated sample gave only a small peak in the chromatogram, corresponding to chloride ions (Cl^−^). Quantitative analyses showed that the intensity of this peak and the corresponding concentration in solution was considerably higher after plasma treatment (Table 1). In addition, because of the plasma treatment, a peak due to nitrate ions (${\text{NO}}_{3}^{-}$) also appeared in the ion chromatogram. ${\text{NO}}_{3}^{-}$ was most likely present due to the interaction of the discharge with residual environmental air, which could be mixed in the solvent during the treatment.

The ion chromatograms of the untreated, argon-streamed and plasma-treated CHCl_3_:DMF mixture are shown in Figure 4B. Similar to pure chloroform, the untreated and argon-streamed samples only showed a small peak corresponding to Cl^−^, while the plasma-treated sample revealed a significantly larger Cl^−^ peak and the formation of nitrate ions. Moreover, compared to plasma-treated chloroform, one additional peak appeared in the chromatogram of the plasma-treated solvent mixture, which could be ascribed to formate (HCOO^−^). Also in this case, the concentration of chloride ions has been quantified (Table 1). Compared to pure treated chloroform, the amount of Cl^−^ was one order of magnitude higher when DMF was added to chloroform. On the contrary, the concentration of nitrates remained more or less the same, confirming the hypothesis that nitrates were formed from residual environmental air in the plasma set-up.

The pH of the aqueous extracts from the organic solvents used in this study was also determined before and after plasma treatment. The results, summarized in Table 1, clearly showed a decrease of the solution pH induced by plasma treatment. The observed increased acidity could be directly linked to the increased concentration of chloride and to the formation of nitrate and formate ions, considering that these species were produced in their acidic form.

#### Spin-Trapping Experiments

Spin-trapping experiments have been performed to detect and identify radicals in solution. A spin trap is a diamagnetic compound (most commonly an organic nitroso or nitrone compound) that can react with a radical species to form a paramagnetic adduct with a lifetime long enough to be detected by EPR spectroscopy (Alberti and Macciantelli, 2009). The spin-trapping analysis of the plasma treated solvents has already been presented in detail in a previous work (Rezaei et al., 2018a). Here we supplemented that analysis with some new results obtained by spin-trapping experiments done in pure chloroform.

**Figure 5:**
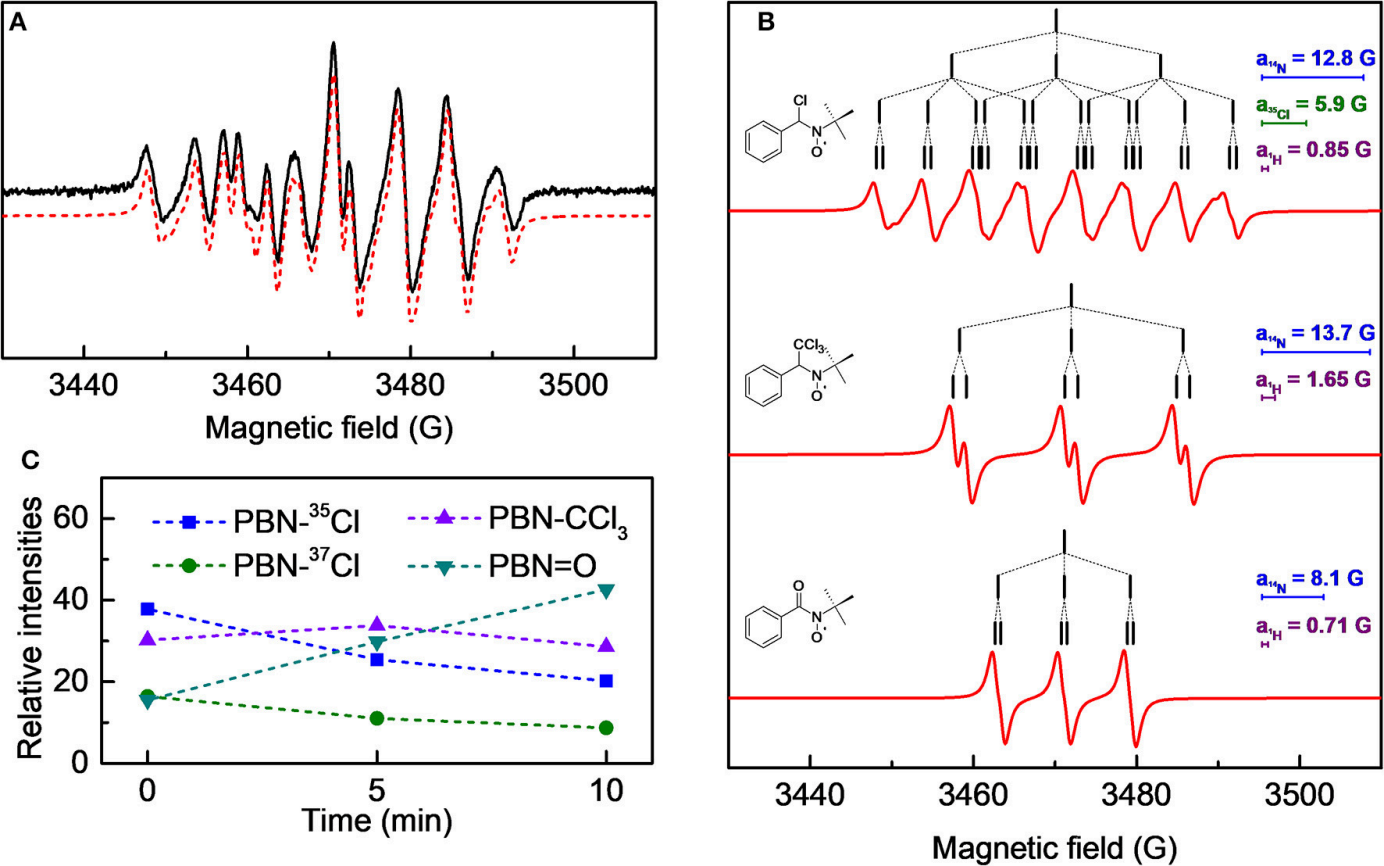
**(A)** cw-EPR spectrum of a 1.0·10^−2^ M PBN solution in chloroform treated with plasma for 3 min and analyzed immediately after the treatment. In red the simulated spectrum, obtained as sum of the three species as reported in **(B)**; the stick-plots in the figure insets report the multiplet structure of the EPR components. **(C)** Time evolution of the composition of the cw-EPR spectrum.

A 1.0·10^−2^ M chloroform solution of spin-trap PBN (N-tert-Butyl-α-phenylnitrone) was treated in the plasma reactor for the desired time and, immediately after the treatment, analyzed by EPR. As an example, the cw-EPR spectrum of a solution treated with plasma for 3 min is reported in Figure 5A. The careful reproduction of the experimental spectrum by means of an appropriate simulation software (Duling, 1994) revealed that the spectrum is the sum of different contributions, as presented in Figure 5B, with the relative hyperfine interaction values (*a*_i_) which are also summarized in Table 2. Comparison of these hyperfine values with data reported in the literature (Davies and Slater, 1986) enabled us to identify the various components (see the first column of Table 2, and the attributed structures in Figure 5B). Please note that for the adduct PBN-Cl (formed by trapping a Cl atom), the simulation takes into account the presence of both ^35^Cl and ^37^Cl isotopes in fixed natural abundance (76 and 24%, respectively) (Davies and Slater, 1986); for them, *a*(^37^Cl)/*a*( ^35^Cl) = *g*_*N*_(^37^Cl)/*g*_*N*_(^35^Cl). The acyl nitroxide has been observed in other works (Ohto et al., 1977; Niki et al., 1983) and is attributed to an oxidation product of the spin-trap (Davies and Slater, 1986), likely formed by reaction of PBN with some oxidizing reactive species of the plasma.

**Table 2:** Hyperfine coupling constants for the relative nuclei of the contributions displayed in Figure 5B used for the simulation of spectrum Figure 5A.

**Detected radical**	***a_***N***_* (G)**	***a_***H***_* (G)**	***a_***Cl***_* (G)**
PBN-^35^Cl	12.8	0.85	6.2
PBN-^37^Cl	12.8	0.85	5.2
PBN-CCl_3_	13.7	1.65	0
PBN = O	8.1	0.71	–

We followed the time evolution of the cw-EPR spectrum, by acquiring spectra at different delay times after the end of the treatment. No further species were observed, but the relative weight of the three species changed in time, as reported in Figure 5C. Specifically, the signals due to the PBN-CCl_3_ and PBN-Cl adducts decreased in time whereas that assigned to the PBN = O adduct increased. The overall intensity did not significantly change in 10 min, but a substantial decay of all adducts intensities was observed after 20 min. Possibly the PBN = O adduct was produced because of exposure to air, but we cannot exclude other mechanisms of production. For instance, a similar rise of the PBN oxidation products has been found for a system in which Cl radicals were produced (Callison et al., 2012). In that case the authors invoked as a possible mechanism the reaction of PBN with relatively long lived molecular chlorine, produced from atomic chlorine.

## Discussion

Considering all the species detected using the various techniques employed in this study, it is possible to outline the major chemical processes taking place when the argon plasma jet is applied to chloroform and to chloroform containing 10% DMF. It is reasonable to assume that these chemical processes occur in the argon plasma bubbles where chloroform and dimethylformamide will also be present as gases due to the evaporation induced by the discharge. The stable products formed within the gas phase are then transferred into the liquid. The formation of chloroethanes is attributed to radical recombination reactions, specifically two ·CCl_3_ radicals in case of hexachloroethane (Shilov and Sabirova, 1959; Michael et al., 1993), one ·CCl_3_ and one ·CHCl_2_ radical in case of pentachloroethane and two ·CHCl_2_ radicals in case of 1,1,2,2-tetrachloroethane, as shown in reactions 1–3. Perchloroethane, Cl_3_C-CCl_3_, was the most abundant chloroethane detected by GC-MS analysis in plasma-treated chloroform. The ·CCl_3_ radical was indeed observed by EPR spectroscopy in a freshly plasma treated sample of pure chloroform. In contrast, Cl_2_HC-CHCl_2_ was barely detectable in the GC-MS chromatogram suggesting that the ·CHCl_2_ radical was present in lower concentration than ·CCl_3_. This conclusion is consistent with the fact that this radical was not observed by EPR analysis.

(1) $$\begin{array}{l} 2\cdot \text{CC}{\text{l}}_{3}\to \text{C}{\text{l}}_{3}\text{C}-\text{CC}{\text{l}}_{3} \end{array}$$

(2) $$\begin{array}{l} \cdot \text{CC}{\text{l}}_{3}+\cdot \text{CHC}{\text{l}}_{2}\to \text{C}{\text{l}}_{3}\text{C}-\text{CHC}{\text{l}}_{2} \end{array}$$

(3) $$\begin{array}{l} 2\cdot \text{CHC}{\text{l}}_{2}\to \text{C}{\text{l}}_{2}\text{HC}-\text{CHC}{\text{l}}_{2} \end{array}$$

The formation of tetrachloroethene can be ascribed to the recombination of two :CCl_2_ carbene units (4) (Won and Bozzelli, 1992).

(4) $$\begin{array}{l} 2:\text{CC}{\text{l}}_{2}\to \text{C}{\text{l}}_{2}\text{C}=\text{CC}{\text{l}}_{2} \end{array}$$

According to the literature, the formation of :CCl_2_ from chloroform can occur either via loss of ·H from ·CHCl_2_ (5) (Won and Bozzelli, 1992) or via chloroform dissociation into :CCl_2_ and HCl (6) (Semeluk and Bernstein, 1954). The formation of hydrogen chloride was indeed verified experimentally by the measurement of the solution pH (H^+^) and ion chromatography (Cl^−^).

(5) $$\begin{array}{l} \cdot \text{CHC}{\text{l}}_{2}\to \cdot \text{H}+:\text{CC}{\text{l}}_{2} \end{array}$$

(6) $$\begin{array}{l} \text{CHC}{\text{l}}_{3}\to :\text{CC}{\text{l}}_{2}+\text{HCl} \end{array}$$

In the literature :CCl_2_ and HCl are reported to be the main products of chloroform thermal decomposition (Semeluk and Bernstein, 1954; Chuang and Bozzelli, 1986) but also of its decomposition induced by non-thermal plasma (Foglein et al., 2005; Gaikwad et al., 2013). In the case of the APPJ used in this work, thermal dissociation is highly unlikely because the gas temperature was too low. We thus believe that chloroform/electron interactions are responsible for the formation of :CCl_2_ and HCl.

Chloroform thermal decomposition (Semeluk and Bernstein, 1954) or chloroform excitation by interaction with electrons, photons or Ar metastables (Yang et al., 1994) can also induce the homolytic dissociation of a C-Cl bond (7), and, less likely, of the C-H bond (8). We succeeded in detecting two of the radicals formed in these reactions, notably ·Cl and ·CCl_3_, by spin trapping and EPR analysis. Evidence for the formation of the third, ·CHCl_2_, was provided by the observation of Cl_3_C-CHCl_2_ and Cl_2_HC-CHCl_2_ among the products of plasma treatment. Failure to detect CHCl_2_ by EPR analysis could be attributed to its low concentration in the system.

(7) $$\begin{array}{l} {\text{CHCl}}_{3}\overset{{\text{e}}^{-}\text{ or h}\nu \text{ or }{\text{Ar}}^{*}}{\to }\cdot \text{Cl}+\cdot {\text{CHCl}}_{2} \end{array}$$

(8) $$\begin{array}{l} {\text{CHCl}}_{3}\overset{{\text{e}}^{-}\text{ or h}\nu \text{ or }{\text{Ar}}^{*}}{\to }\cdot \text{H}+\cdot {\text{CCl}}_{3} \end{array}$$

Reaction (8) is less probable than reaction (7) due to the higher dissociation energy of the C-H bond with respect to the C-Cl bond [average bond dissociation energies for C-H and C-Cl bonds are 4.13 eV e 3.43 eV, respectively (Weissman and Benson, 1983)]. Thus, an alternative source of ·CCl_3_ must be considered to account for its higher abundance than ·CHCl_2_, specifically the reaction of ·Cl with chloroform, which proceeds via hydrogen abstraction (9) (Orlando, 1999).

(9) $$\begin{array}{l} \cdot \text{Cl}+\text{CHC}{\text{l}}_{3}\to \cdot \text{CC}{\text{l}}_{3}+\text{HCl} \end{array}$$

When DMF was added to chloroform and the mixture CHCl_3_:DMF 9:1 was subjected to plasma treatment, two significant changes in the product distribution were observed: the concentration of Cl^−^ increased by a 10-fold factor and Cl_3_C-CCl_3_ was no longer the most abundant chloroethane produced, the area of its chromatographic peak becoming similar to those of Cl_3_C-CHCl_2_ and Cl_2_HC-CHCl_2_. The latter observation implies that in the mixed solvent the ·CHCl_2_ radical was formed in similar concentration as ·CCl_3_. All these observations are rationalized if one considers that in the presence of DMF another important process takes place, i.e., dissociative electron attachment to chloroform (10). The products of this reaction are indeed chloride and ·CHCl_2_, as known from literature (Scheunemann et al., 1980; Matejcik et al., 1997). The promotion of this process in the presence of DMF could be attributed to the well-known ability of DMF to solvate and stabilize ions, especially anions.

(10) $$\begin{array}{l} \text{CHC}{\text{l}}_{3}+{\text{e}}^{-}\to \text{C}{\text{l}}^{-}+\cdot \text{CHC}{\text{l}}_{2} \end{array}$$

Thus, reaction (10) accounts for both the increase of recombination products Cl_3_C-CHCl_2_ and Cl_2_HC-CHCl_2_ with respect to Cl_3_C-CCl_3_ and the increase of chloride ions observed in the mixture CHCl_3_:DMF 9:1 with respect to pure CHCl_3_. It cannot be excluded that dissociative electron attachment may also occur in the liquid phase involving solvated electrons, formed at the gas/liquid interface and reacting there or within the solvent in the first layers in contact with the gas. It is known, indeed, that solvated electrons undergo efficient dissociative electron attachment reactions with chlorinated organic compounds in aqueous media producing chloride (Lichtscheidl and Getoff, 1976; Naik and Mohan, 2005; Yuan et al., 2015) and that they are also involved in organic solvents.

Two additional species observed in the presence of DMF are N-methylformamide and formic acid. N-methylformamide may originate from thermal or electron induced decomposition of DMF via homolytic dissociation of the N-C bond (11a), followed by hydrogen abstraction from chloroform (11b). As for formic acid, we believe it formed via hydrolysis of DMF which can occur in the presence of traces of water with acid catalysis. The presence of traces of water was previously detected by the appearance of the OH signal in the emission spectroscopy spectrum acquired during the plasma treatment of the mixture CHCl_3_:DMF 9:1 (Grande et al., 2017).

(11a) $$\begin{array}{l} \text{HC}\left(=\text{O}\right)\text{N}{\left(\text{C}{\text{H}}_{3}\right)}_{2}+{\text{e}}^{-}\to \text{HC}\left(=\text{O}\right)\text{N}\cdot \left(\text{C}{\text{H}}_{3}\right)+\cdot \text{C}{\text{H}}_{3}+{\text{e}}^{-} \end{array}$$

(11b) $$\begin{array}{l} \text{HC}\left(=\text{O}\right)\text{N}\cdot \left(\text{C}{\text{H}}_{3}\right)+\text{CHC}{\text{l}}_{3}\to \text{HC}\left(=\text{O}\right)\text{NH}\left(\text{C}{\text{H}}_{3}\right)+\cdot \text{CC}{\text{l}}_{3} \end{array}$$

Finally, another process which must be considered is the ionization of the solvent. Considering the lower ionization energy of chloroform (EI = 11.37 eV) and of DMF (EI = 9.13 eV) with respect to that of argon (EI = 15.76 eV) (Linstrom and Mallard, 2019), it is expected that both solvents undergo ionization via charge exchange with argon ions (12a and 13a). The resulting radical cations can dissociate leading to ${\text{CHCl}}_{2}^{+}$ and ·Cl (12b) and to HC(= O)N^+^(CH_3_) and ·CH_3_ (13b), respectively.

(12a) $$\begin{array}{l} \text{CHC}{\text{l}}_{3}+\text{Ar}\cdot^{+}\to \text{CHC}{\text{l}}_{3}\cdot^{+}+\text{Ar} \end{array}$$

(12b) $$\begin{array}{l} \text{CHC}{\text{l}}_{3}\cdot^{+}\to \text{CHC}{\text{l}}_{2}^{+}+\cdot \text{Cl} \end{array}$$

(13a) $$\begin{array}{l} \text{HC}\left(=\text{O}\right)\text{N}{\left(\text{C}{\text{H}}_{3}\right)}_{2}+\text{Ar}\cdot^{+}\to \text{HC}\left(=\text{O}\right)\text{N}{\left(\text{C}{\text{H}}_{3}\right)}_{2}\cdot^{+}+\text{ Ar} \end{array}$$

(13b) $$\begin{array}{l} \text{HC}\left(=\text{O}\right)\text{N}{\left(\text{C}{\text{H}}_{3}\right)}_{2}\cdot^{+}\to \text{HC}\left(=\text{O}\right){\text{N}}^{+}\left(\text{C}{\text{H}}_{3}\right)+\cdot \text{C}{\text{H}}_{3} \end{array}$$

Since the proton affinity of CCl_2_ (8.92 eV) is lower than that of DMF (9.19 eV) (Hunter and Lias, 1998), proton transfer from ${\text{CHCl}}_{2}^{+}$ to DMF in the gas phase is thermodynamically favored and expected to be kinetically very fast (14). It is worth noting that reaction (14) may thus contribute to the acidification of the solution and provide a direct entry, in the presence of water traces, to acid catalyzed hydrolysis of DMF.

(14) $${\text{CHCl}}_{2}^{+}+\text{HC}(=\text{O})\text{N}{\left({\text{CH}}_{3}\right)}_{2}\to :{\text{CCl}}_{2}+\text{HC}(=\text{O}){\text{NH}}^{+}{\left({\text{CH}}_{3}\right)}_{2}$$

## Conclusions

All the experimental results obtained in the work described here fit nicely into a coherent mechanistic picture. We can thus compare and rationalize the effects of plasma treatment of CHCl_3_ and of the CHCl_3_:DMF 9:1 mixture, which are useful solvents for the production of nanofibers by electrospinning of polymer solutions. It was found that plasma induces the formation of hydrogen chloride, a process which is more pronounced in the CHCl_3_/DMF solvent mixture than in pure CHCl_3_. The detection of tetrachloroethene and of chloroethanes, the products of recombination of :CCl_2_, ·CCl_3_ and ·CHCl_2_ radicals, allowed us to identify the major reaction pathways of chloroform in the absence and in the presence of DMF and, specifically, to underline the prominent role of DMF in the global process. These findings are valuable *per se*, considering the present lack of data and knowledge on the interaction of non-thermal plasma with organic solvents and on its consequences. They also explain the beneficial effect on PCL electrospinning, observed when the solvent, a CHCl_3_:DMF 9:1 mixture, was preliminarily treated by plasma prior to the addition of the polymer. It is thus proven that pretreatment of the solvent is an interesting possibility for electrospinning and it is expected that other applications might take advantage of this novel approach.

## Data Availability

All datasets generated for this study are included in the manuscript and/or the supplementary files.

## Author Contributions

ND and RM conceived the research project. SG, FT, AN, AG, AB, and EM designed and performed the experiments and analyzed the data. SG, FT, AB, CP, and EM wrote the paper. All authors discussed the results and revised the paper.

### Conflict of Interest Statement

The authors declare that the research was conducted in the absence of any commercial or financial relationships that could be construed as a potential conflict of interest.

## References

[B1] Alberti A. Macciantelli D. (2009). Spin trapping, in Electron Paramagnetic Resonance: A Practitioner's Toolkit, eds Brustolon M. R. Giamello E. (Hoboken, NJ: Wiley), 287–323.

[B2] Bhardwaj N. Kundu S. C. (2010). Electrospinning: a fascinating fiber fabrication technique. Biotechnol. Adv. 28, 325–347. 10.1016/j.biotechadv.2010.01.004 20100560

[B3] Bruggeman P. Leys C. (2009). Non-thermal plasmas in and in contact with liquids. J. Phys. D. Appl. Phys. 42:053001 10.1088/0022-3727/42/5/053001

[B4] Bruggeman P. J. Kushner M. J. Locke B. R. Gardeniers J. G. E. Graham W. G. Graves D. B. (2016). Plasma-liquid interactions: a review and roadmap. Plasma Sources Sci. Technol. 25:053002 10.1088/0963-0252/25/5/053002

[B5] Callison J. Edge R. De Cuba K. R. Carr R. H. McDouall J. J. W. Collison D. (2012). Origin of impurities formed in the polyurethane production chain. 1. Conditions for chlorine transfer from an aryl isocyanide dichloride byproduct. Ind. Eng. Chem. Res. 51, 2515–2523. 10.1021/ie2013136

[B6] Chuang S. C. Bozzelli J. W. (1986). Conversion of chloroform to hydrochloric acid by reaction with hydrogen and water vapor. Environ. Sci. Technol. 20, 568–574. 10.1021/es00148a004 19994952

[B7] Colombo V. Fabiani D. Focarete M. L. Gherardi M. Gualandi C. Laurita R. (2014). Atmospheric pressure non-equilibrium plasma treatment to improve the electrospinnability of poly(L -lactic acid) polymeric solution. Plasma Process. Polym. 11, 247–255. 10.1002/ppap.201300141

[B8] Davies M. J. Slater T. F. (1986). Electron spin resonance spin trapping studies on the photolytic generation of halocarbon radicals. Chem. Biol. Interact. 58, 137–147. 10.1016/S0009-2797(86)80093-X 3013435

[B9] Duling D. R. (1994). Simulation of multiple isotropic Spin-Trap EPR spectra. J. Magn. Reson. Ser. B 104, 105–110. 10.1006/jmrb.1994.1062 8049862

[B10] Foglein K. A. Szabó P. T. Babievskaya I. Z. Szépvölgyi J. (2005). Comparative study on the decomposition of chloroform in thermal and cold plasma. Plasma Chem. Plasma Process. 25, 289–302. 10.1007/s11090-004-3041-y

[B11] Frenot A. Chronakis I. S. (2003). Polymer nanofibers assembled by electrospinning. Curr. Opin. Colloid Interface Sci. 8, 64–75. 10.1016/S1359-0294(03)00004-9

[B12] Gaikwad V. Kennedy E. Mackie J. Holdsworth C. Molloy T. Kundu S. (2013). Reaction of chloroform in a non-oxidative atmosphere using dielectric barrier discharge, in Digest of Technical Papers-IEEE International Pulsed Power Conference (San Francisco, CA), 6627401 10.1109/PPC.2013.6627401

[B13] Grande S. Van Guyse J. Nikiforov A. Y. Onyshchenko I. Asadian M. Morent R. (2017). Atmospheric pressure plasma jet treatment of poly-ϵ-caprolactone polymer solutions to improve electrospinning. ACS Appl. Mater. Interfaces 9, 33080–33090. 10.1021/acsami.7b08439 28871776

[B14] Hsu C. M. Shivkumar S. (2004). N,N-dimethylformamide additions to the solution for the electrospinning of poly(ε-caprolactone) nanofibers. Macromol. Mater. Eng. 289, 334–340. 10.1002/mame.200300224

[B15] Huang J. Virji S. Weiller B. H. Kaner R. B. (2003). Polyaniline nanofibers: facile synthesis and chemical sensors. J. Am. Chem. Soc. 125, 314–315. 10.1021/ja028371y 12517126

[B16] Hunter E. P. L. Lias S. G. (1998). Evaluated gas phase basicities and proton affinities of molecules: an update. J. Phys. Chem. Ref. Data 27, 413–656. 10.1063/1.556018

[B17] Lichtscheidl J. Getoff N. (1976). Radiolysis of halogenated aromatic compounds in aqueous solutions—I conductometric pulse radiolysis and steady-state studies of the reaction of ${\text{e}}_{\text{aq}}^{-}$. Int. J. Radiat. Phys. Chem. 8, 661–665. 10.1016/0020-7055(76)90037-1

[B18] Linstrom P. J. Mallard W. G (eds) (2019). NIST Chemistry WebBook, NIST Standard Reference Database Number 69. Gaithersburg, MD: National Institute of Standards and Technology 10.18434/T4D303.

[B19] Long Y. Z. Yu M. Sun B. Gu C. Z. Fan Z. (2012). Recent advances in large-scale assembly of semiconducting inorganic nanowires and nanofibers for electronics, sensors and photovoltaics. Chem. Soc. Rev. 41, 4560–4580. 10.1039/c2cs15335a 22573265

[B20] Matejcik S. Senn G. Scheier P. Kiendler A. Stamatovic A. Märk T. D. (1997). Dissociative electron attachment cross section to CHCl3 using a high resolution crossed beams technique. J. Chem. Phys. 107, 8955–8962. 10.1063/1.475187

[B21] Michael J. V. Lim K. P. Kumaran S. S. Kiefer J. H. (1993). Thermal decomposition of carbon tetrachloride. J. Phys. Chem. 97, 1914–1919. 10.1021/j100111a032

[B22] Naik D. B. Mohan H. (2005). Radiolysis of aqueous solutions of dihalobenzenes: studies on the formation of halide ions by ion chromatography. Radiat. Phys. Chem. 73, 218–223. 10.1016/j.radphyschem.2004.08.010

[B23] Niki E. Yokoi S. Tsuchiya J. Kamiya Y. (1983). Spin trapping of peroxy radicals by phenyl N-(tert-butyl) nitrone and methyl N-duryl nitrone. J. Am. Chem. Soc. 105, 1498–1503. 10.1021/ja00344a014

[B24] Ohto N. Niki E. Kamiya Y. (1977). Study of autoxidation by spin trapping. Spin trapping of peroxyl radicals by phenyl N-t-butyl nitrone. J. Chem. Soc. Perkin Trans. 2, 1770–1774. 10.1039/p29770001770

[B25] Orlando J. J. (1999). Temperature dependence of the rate coefficients for the reaction of chlorine atoms with chloromethanes. Int. J. Chem. Kinet. 31, 515–524. 10.1002/(SICI)1097-4601(1999)31:7<515::AID-KIN6>3.0.CO;2-1

[B26] Qin X. H. Yang E. L. Li N. Wang S. Y. (2007). Effect of different salts on electrospinning of polyacrylonitrile (PAN) polymer solution. J. Appl. Polym. Sci. 103, 3865–3870. 10.1002/app.25498

[B27] Reneker D. H. Yarin A. L. (2008). Electrospinning jets and polymer nanofibers. Polymer 49, 2387–2425. 10.1016/j.polymer.2008.02.002

[B28] Rezaei F. Gorbanev Y. Chys M. Nikiforov A. Van Hulle S. W. H. Cos P. (2018a). Investigation of plasma-induced chemistry in organic solutions for enhanced electrospun PLA nanofibers. Plasma Process. Polym. 15:1700226 10.1002/ppap.201700226

[B29] Rezaei F. Nikiforov A. Morent R. De Geyter N. (2018b). Plasma modification of poly lactic acid solutions to generate high quality electrospun PLA nanofibers. Sci. Rep. 8:2241. 10.1038/s41598-018-20714-5 29396416 PMC5797159

[B30] Ryu S. Y. Kwak S. Y. (2013). Role of electrical conductivity of spinning solution on enhancement of electrospinnability of polyamide 6,6 nanofibers. J. Nanosci. Nanotechnol. 13, 4193–4202. 10.1166/jnn.2013.5863 23862472

[B31] Scheunemann H.-U. Illenberger E. Baumgärtel H. (1980). Dissociative electron attachment to CCl4, CHCl3, CH2Cl2 and CH3Cl. Berichte Bunsengesellschaft Phys. Chem. 84, 580–585. 10.1002/bbpc.19800840612

[B32] Semeluk G. P. Bernstein R. B. (1954). The thermal decomposition of chloroform. I. products 1a. J. Am. Chem. Soc. 76, 3793–3796. 10.1021/ja01643a060

[B33] Shabafrooz V. Mozafari M. Vashaee D. Tayebi L. (2014). Electrospun nanofibers: from filtration membranes to highly specialized tissue engineering scaffolds. J. Nanosci. Nanotechnol. 14, 522–534. 10.1166/jnn.2014.9195 24730280

[B34] Shi Q. Vitchuli N. Nowak J. Lin Z. Guo B. McCord M. (2011). Atmospheric plasma treatment of pre-electrospinning polymer solution: a feasible method to improve electrospinnability. J. Polym. Sci. Part B Polym. Phys. 49, 115–122. 10.1002/polb.22157

[B35] Shilov A. E. Sabirova R. D. (1959). Mechanism of the primary thermal breakdown of chlorine derivatives of methane. I. Breakdown of carbon tetrachloride. Zh. Fiz. Khim. 33:1365.

[B36] Šunka P. Babický V. Clupek M. Lukeš P. Šimek M. Schmidt J. (1999). Generation of chemically active species by electrical discharges in water. Plasma Sources Sci. Technol. 8, 258–265. 10.1088/0963-0252/8/2/006

[B37] Teo W. E. Ramakrishna S. (2006). A review on electrospinning design and nanofibre assemblies. Nanotechnology 17, R89–R106. 10.1088/0957-4484/17/14/R01 19661572

[B38] Venugopal J. Ramakrishna S. (2005). Applications of polymer nanofibers in biomedicine and biotechnology. Appl. Biochem. Biotechnol. Part A Enzym. Eng. Biotechnol. 125, 147–157. 10.1385/ABAB:125:3:147 15917579

[B39] Weissman M. Benson S. W. (1983). Heat of formation of the CHCl2 radical. Bond dissociation energies in chloromethanes and chloroethanes. J. Phys. Chem. 87, 243–244. 10.1021/j100225a014

[B40] Won Y. S. Bozzelli J. W. (1992). Chloroform pyrolysis: experiment and detailed reaction model. Combust. Sci. Technol. 85, 345–373. 10.1080/00102209208947177

[B41] Yang X. Felder P. Robert Huber J. (1994). Photodissociation of the CHFCl2 and CHCl3 molecules and the CHCl2 radical in a beam at 193 nm. Chem. Phys. 189, 127–136. 10.1016/0301-0104(94)80012-X

[B42] Yuan H. Pan H. Shi J. Li H. Dong W. (2015). Kinetics and mechanisms of reactions for hydrated electron with chlorinated benzenes in aqueous solution. Front. Environ. Sci. Eng. 9, 583–590. 10.1007/s11783-014-0691-8

[B43] Zong X. Kim K. Fang D. Ran S. Hsiao B. S. Chu B. (2002). Structure and process relationship of electrospun bioabsorbable nanofiber membranes. Polymer 43, 4403–4412. 10.1016/S0032-3861(02)00275-6

